# Supplementary material for: Impact of environmental factors in predicting daily severity scores of atopic dermatitis
Source: Clin Transl Allergy. 2021 Apr 28;11(2):e12019. doi: 10.1002/clt2.12019 (PMC8099209; doi:10.1002/clt2.12019)
Supplement: Supplementary file 1 — Supplementary Material 1 [file CLT2-11-e12019-s001.pdf]

Impact of environmental factors in predicting daily severity scores of atopic dermatitis

Guillem Hurault[1], Valentin Delorieux[1], Young-Min Kim[2], Kangmo Ahn[2], Hywel C. Williams[3], Reiko J. Tanaka[1]

1 Department of Bioengineering, Imperial College London, UK

2 Department of Pediatrics, Samsung Medical Center, Sungkyunkwan University School of Medicine; Environmental Health Centre for Atopic diseases, Samsung Medical Center, Korea

3 Centre of Evidence-Based Dermatology, University of Nottingham, UK

Supporting Information Text

The probability mass function of the ordered logistic distribution for an outcome $y \in \{0,1,2,3,4\}$ is parametrised by a location η and a vector of cut-off value, $\boldsymbol{c} = (c_0 \; c_1 \; c_2 \; c_3)$, $c_0 < c_1 < c_2 < c_3$, as

$$\text{OrderedLogistic}(y \mid \eta, \boldsymbol{c}) = \begin{cases} 1 - \text{logit}^{-1}(\eta - c_0), & \text{if } y = 0, \\ \text{logit}^{-1}(\eta - c_{y-1}) - \text{logit}^{-1}(\eta - c_y), & \text{if } y = 1, 2, 3, \\ \text{logit}^{-1}(\eta - c_3), & \text{if } y = 4. \end{cases}$$

In practice, the logits can be obtained by jointly fitting the cumulative distribution, $P(y \leq 0)$, $P(y \leq 1)$, $P(y \leq 2)$ and $P(y \leq 3)$, with logistic regressions.

Supporting Figures

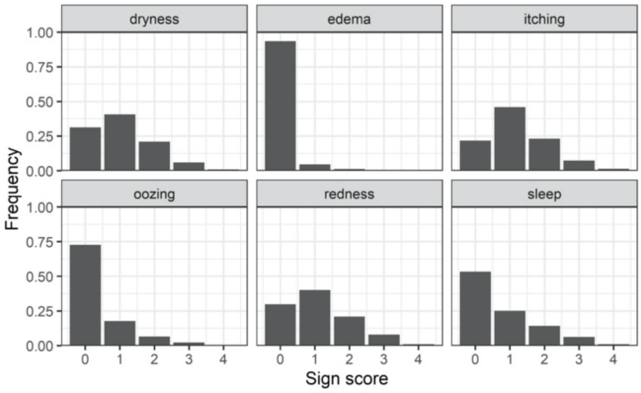

Figure S1: Distribution of the AD signs scores across time and patients.

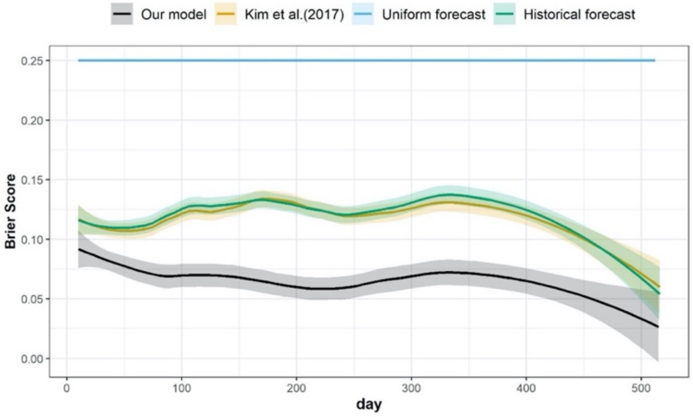

Figure S2: Comparison of the predictive performance for the models predicting the AD symptom state. Our model (without covariate and for which the prediction for the AD symptom state is derived from the predictions for each AD sign) is compared to the uniform and the historical forecast models, and the logistic regression model proposed in Kim et al. (2017) [10]. *The performance is measured by the Brier score (the lower Brier score corresponds to the better predictive performance). Learning curves were obtained using LOWESS smoothing. Shaded areas correspond to* $\pm$ 1.96 *standard error.*
